# HIV-1 Tat amino acid residues that influence Tat-TAR binding affinity: a scoping review

**DOI:** 10.1186/s12879-023-08123-0

**Published:** 2023-03-17

**Authors:** Piwai Terry Gotora, Rencia van der Sluis, Monray Edward Williams

**Affiliations:** Human Metabolomics, North-West University, Potchefstroom, South Africa

**Keywords:** Tat polymorphism, Transactivation of transcription, Transactivation response RNA element, Subtype variation, Molecular binding

## Abstract

HIV-1 remains a global health concern and to date, nearly 38 million people are living with HIV. The complexity of HIV-1 pathogenesis and its subsequent prevalence is influenced by several factors including the HIV-1 subtype. HIV-1 subtype variation extends to sequence variation in the amino acids of the HIV-1 viral proteins. Of particular interest is the transactivation of transcription (Tat) protein due to its key function in viral transcription. The Tat protein predominantly functions by binding to the transactivation response (TAR) RNA element to activate HIV-1 transcriptional elongation. Subtype-specific Tat protein sequence variation influences Tat-TAR binding affinity. Despite several studies investigating Tat-TAR binding, it is not clear which regions of the Tat protein and/or individual Tat amino acid residues may contribute to TAR binding affinity. We, therefore, conducted a scoping review on studies investigating Tat-TAR binding. We aimed to synthesize the published data to determine (1) the regions of the Tat protein that may be involved in TAR binding, (2) key Tat amino acids involved in TAR binding and (3) if Tat subtype-specific variation influences TAR binding. A total of thirteen studies met our inclusion criteria and the key findings were that (1) both N-terminal and C-terminal amino acids outside the basic domain (47–59) may be important in increasing Tat-TAR binding affinity, (2) substitution of the amino acids Lysine and Arginine (47–59) resulted in a reduction in binding affinity to TAR, and (3) none of the included studies have investigated Tat subtype-specific substitutions and therefore no commentary could be made regarding which subtype may have a higher Tat-TAR binding affinity. Future studies investigating Tat-TAR binding should therefore use full-length Tat proteins and compare subtype-specific variations. Studies of such a nature may help explain why we see differential pathogenesis and prevalence when comparing HIV-1 subtypes.

**Supplementary Information:**

The online version contains supplementary material available at 10.1186/s12879-023-08123-0.

## Introduction

Human immunodeficiency virus (HIV) is a retrovirus that functions by integrating a copy of its retroviral deoxyribonucleic acid (DNA) genome into the DNA of the infected human cell for replication [1]. HIV was discovered in the early 1980s when the virus had already established a pandemic [2]. HIV-1 is responsible for the development of Acquired Immune Deficiency Syndrome (AIDS) [3]. Since the 1980s to date, about 74.9 million people have been infected by HIV, with 32 million people succumbing to AIDS (the stage defined as a decrease in CD4^+^ cells below 200 cells/µl) [4]. In 2021, an estimated 38.4 million people were recorded to be living with HIV worldwide, with 650,000 deaths and 1.5 million new infections [5]. Adding to this, the coronavirus disease 2019 (COVID-19) pandemic had negatively influenced the progression of HIV. In South Africa, HIV-1 testing, and anti-retroviral therapy (ART) initiations were heavily impacted [6]. Further, the shortage of anti-retroviral drugs (ARVs) due to the shutdowns of certain drug manufacturers [7] and the disruption in the delivery of HIV care due to the COVID-19 pandemic may have increased morbidity and mortality among people living with HIV (PLWH) [8]. The diversion of more healthcare workers to care for COVID-19 patients may also have contributed to an increase in the prevalence of HIV infections and disease progression [9–11]. In both severe acute respiratory syndrome coronavirus type 2 (SARS-CoV-2) and HIV, evolutionary strategies are at play [8] which influences the change in the genetic makeup of these viruses.

Two main types of HIV exist, namely HIV-1 and HIV-2, of which HIV-1 predominates worldwide [9]. HIV-1 is classified into three genetic groups, M, O and N [10]. Most HIV-1 infections worldwide are caused by the group M subtypes which are designated by letters A, B, C, D, F, G, H, J and K [10]. HIV-1 subtypes, also known as clades, are linked geographically or epidemiologically [11]. Subtype C is the most prevalent HIV strain and is the predominant subtype in India and Southern Africa [12]. Subtype B is prevalent in almost all parts of Europe and the Americas, while a diverse variety of subtypes are found in West and Central Africa [13]. Statistically, subtype C represents approximately 50% of the world’s HIV infected while the second most prevalent subtype B accounts for about 12% of PLWH [12, 14]. Subtype D is prevalent in East and Central Africa, with sporadic cases observed in Southern and Western Africa [15]. Collectively, the subtypes F, H, J and K account for 0.94% of all global infections [16]. Subtypes H, J and K are found in Central, Southern and West Africa [17], with subtype K, in particular, being identified in the Democratic Republic of Congo and Cameroon [18]. Subtypes G and A viruses have been identified in western and eastern Africa and also in central Europe [19, 20]. Subtype F is endemic in South and South-East Asia [21].

The HIV virion has a spherical structure with a diameter of approximately 100–130 nm. Its lipid membrane envelope is derived from the host cell and cellular proteins [22]. The HIV genome contains the retroviral genes *gag, pol,* and *env*. In addition, HIV has six regulatory genes (*tat, rev, nef, vif, vpr, and vpu*) and is therefore considered a “complex” retrovirus [22]. HIV encodes for 15 distinct proteins [23, 24], and these include: structural (matrix, capsid, nucleocapsid, p6, surface and transmembrane), Pol enzymes (protease, reverse transcriptase, integrase), regulatory proteins (Transactivation of transcription (Tat) and regulator of expression of virion proteins (Rev)) and accessory proteins (Negative regulatory factor (Nef), viral infectivity factor (Vif), viral protein R (Vpr) and virus protein U (Vpu)). Of particular interest is the Tat protein due to its multifunctional activity within HIV pathogenesis.

**Fig. 1:**
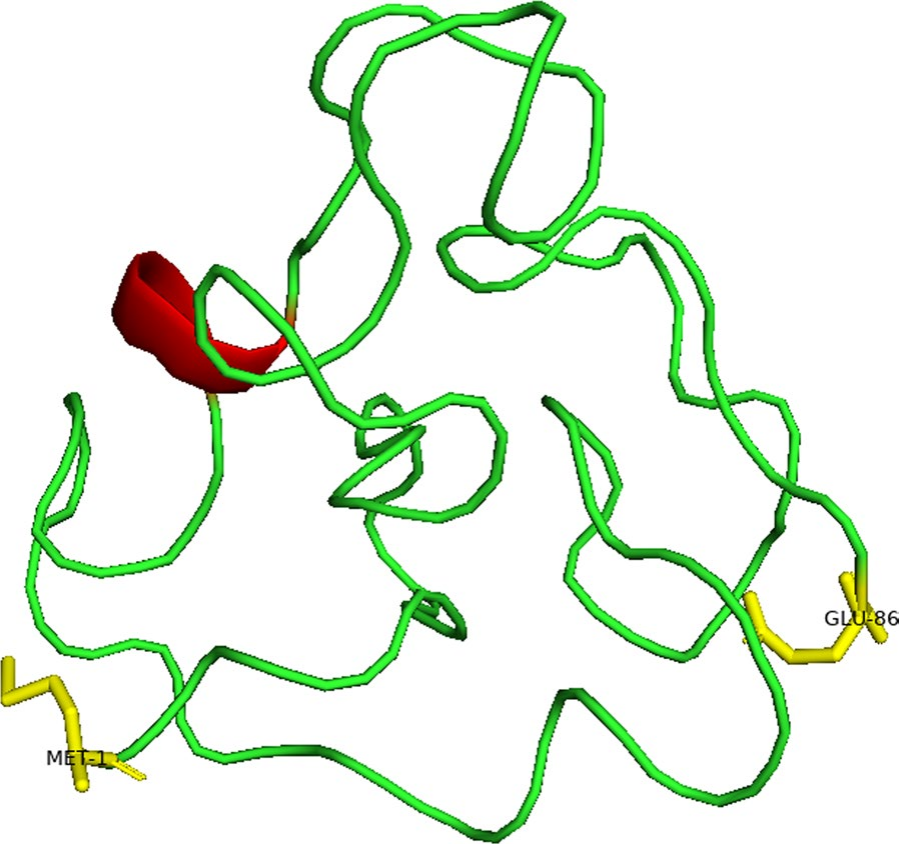
3D predicted structure of the HIV-1 subtype B Tat protein (subtype B, Isolate MN) (1–86) using Swiss-model webserver. The alpha-helical structure is coloured red and the N and C- terminals are yellow coloured

**Fig. 2:**
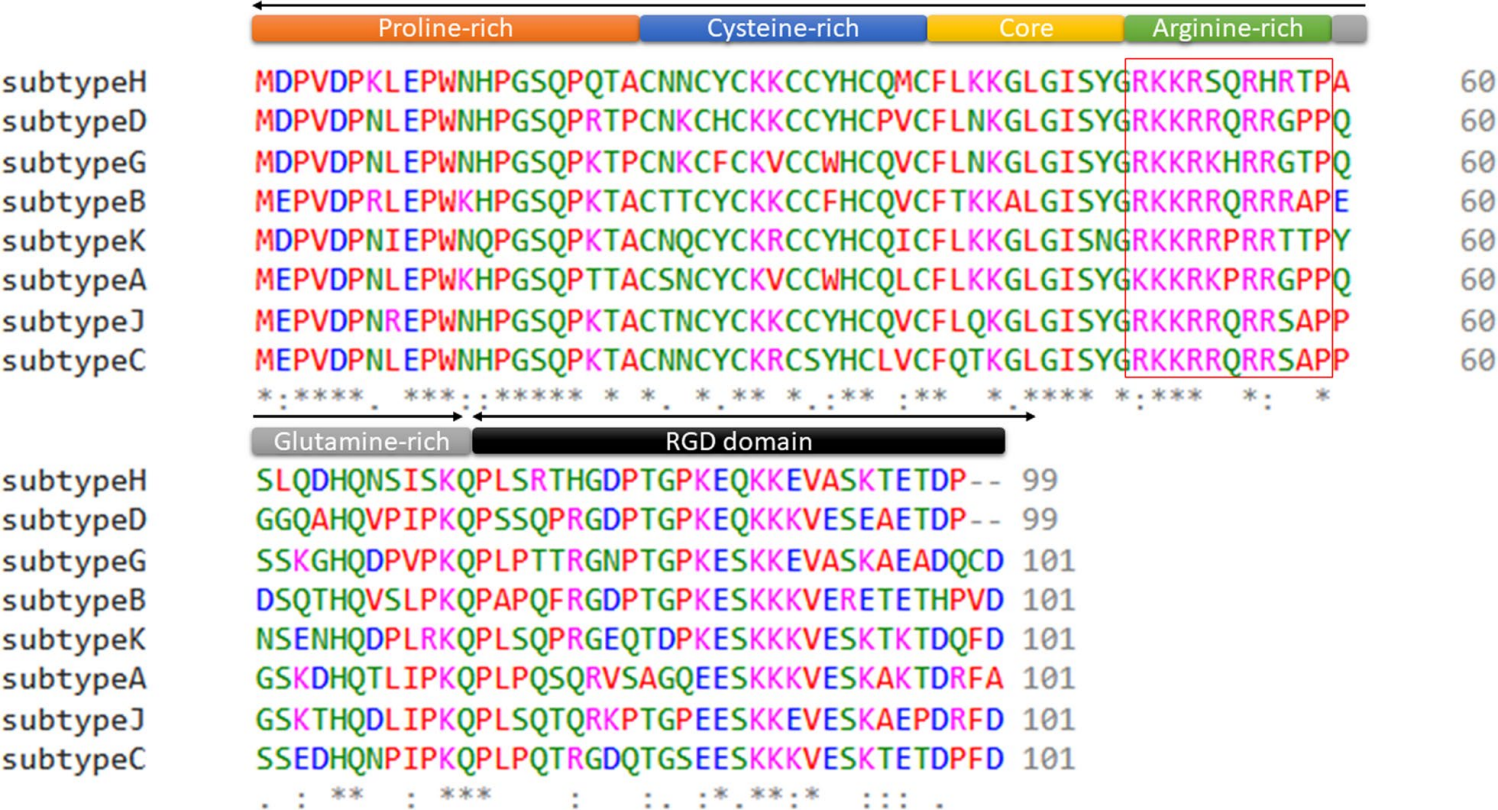
Multiple sequence alignment of various HIV-1 Tat subtypes. From top to bottom; Tat subtype H (isolate 90CF056), subtype D (isolate ELI), subtype G (isolate SE6165), subtype B (isolate MN), subtype K (isolate 96CM-MP535), subtype A (isolate U455), subtype J (isolate SE9280) and subtype C (isolate 92BR025). Tat protein is encoded by two exons, exon one spans the region of amino acids 1–72 and exon two spans the region of 73–101. The Tat protein is made up of six function regions including the proline-rich region (1–21), the cysteine-rich region (22–37), the core region (38–48), the basic, arginine-rich domain (49–59), the glutamine-rich domain (60–72) and the RGD domain (73–101). The black arrows indicate the two exons

HIV-1 Tat is a regulatory protein encoded by the *tat* gene. The Tat protein has a variable weight of 14–16 kilodaltons (kDa), has an amino acid composition varying from 86 to 104 amino acids [25, 26] and is an intrinsically unstructured protein [27] (Figs. 1 and 2). Tat is divided into six different regions and contains various conserved areas within its overall sequence, which are crucial for its function [28]. Region I has a conserved tryptophan (Trp)-11 while the cysteine-rich region II contains seven well-conserved cysteines at positions 22, 25, 27, 30, 31, 34 and 37 (22–37) [29]. In region III (38–48), a conserved Phenylalanine-38 motif Arginine-Lysine-Leucine-Glycine-Isoleucine at 43–48 was observed in HIV-1 subtypes [30]. Region IV, the basic domain (49–59) is the key factor in Tat-TAR binding and is highly conserved among all Tat variants [31, 32]. Region V (60–72) is a glutamine-rich region [33]. This region shows the highest rate of sequence variation [29]. Region VI contains the C-terminus of Tat encoded by the second exon and it shows similarities among Tat variants [34] (Fig. 2).

The various regions/domains of the Tat protein serve specific functions. In particular, the N-terminal (1–48) is considered crucial for the activation of transcription. This is due to the cysteine-rich domain (21–37) being multifunctional and required for dimerization, metal binding, and stabilization of the protein structure [35]. In addition, the hydrophobic core motif (38–48) is critical for transactivation activity and also binds to the RNA TAR element of nascent RNA [36]. The arginine-rich domain (49–59) was shown to be important for Tat localization in the nucleus, binding to the HIV long terminal repeat (LTR) TAR RNAs and Tat internalization into cells by binding to cellular heparan sulphate proteoglycans [37–40]. The glutamine-rich region (60–72) has also been shown to function in TAR interaction and is important for the Tat-apoptosis function [41, 42]. The second exon (73–86) was shown to be less important for transactivation activity, however, studies suggest having a role in virus replication in lymphocytes and macrophages [43–45]. Finally, the C-terminal domain, containing the arginylglycylaspartic acid (RGD) motif is essential for binding and signalling through the same integrin receptors α5β1, αvβ3 and αvβ5, which recognize the RGD region of extracellular matrix proteins [46, 47].

Mutations in the *tat* gene result in variations in its protein amino acid sequence and this may affect Tat function [48]. This can be seen with subtype-specific variation which results in changes to the amino acid sequences within the HIV Tat protein (Fig. 2). Several subtype-specific mutations, specifically the basic Arginine (Arg)-rich region (49–59) exist when comparing the HIV-1 subtypes A, B, C, D, G, H, J and K (Fig. 2). These naturally occurring sequence variations in the Tat protein have been linked to differential pathogenesis [49] and neuropathogenesis [50]. In particular, mutations within the glutamine-rich region (60–72) have been involved in the induction of apoptosis in T-cells [51]. Another study showed that the two-point mutations, L43V and S46F increased the transcriptional activity of Tat and increased its apoptosis induction potential [52]. The naturally occurring glutamate substitution at amino acid 63 that is largely present in subtype C [53] leads to greater transcriptional activity in human CD4 T-cells which are the target of HIV, thus allowing HIV to achieve a high-level of transcription [54]. Further, the Tat substitutions (R57S) have been linked to differential levels of inflammation in cell culture and human studies [40, 55]. Mutations in the Tat protein have also been associated with differential levels of neurotoxicity. An in vivo study has shown that the mutation C22G resulted in significantly less neurotoxicity due to reduced levels of apoptosis [56]. The C22G Tat mutant cannot interact with cyclin-dependent kinase 9 (CDK9) which is critical for RNA Polymerase II (Pol II) transcription initiation and elongation. It is, therefore, transactivation negative [57].

The HIV-1 transactivation response element (TAR) binds to Tat, facilitating viral replication in its latent state [58]. The primary role of Tat is to recognize the 5’-TAR element in the HIV-1 RNA, and its flexible and disorderly structure promotes high-affinity complexes with the RNA [59–61]. Tat recruits the positive transcriptional elongation factor (P-TEFb) onto the nascent viral TAR RNA in order to overcome the elongation pause for activation transcription of the entire viral genome [62]. This elongation factor consists of CDK9 and Cyclin T1. In the absence of Tat, P-TEFb exists in the cell as a large inactive complex composed of 7SK snRNA and MAQ1/HEXIM1 proteins [63, 64]. Once the elongation factor is recruited by Tat to TAR RNA, CDK9 phosphorylates the carboxyl-terminal domain of RNA Polymerase II (RNAP II) and thereby activates elongation [61]. The result of these post-translational modifications is the synthesis of high levels of full-length viral transcripts. Tat is known to interact with multiple host factors that ensure the binding affinity of Tat to TAR, however, for the purpose of this review, we were particularly interested in the major interacting partner which is TAR.

As highlighted above, mutations in the Tat protein influence the pathogenesis and neuropathogenesis of HIV-1. Tat mutations may also influence Tat-TAR binding and subsequent viral transcription. Of the two important functional domains of HIV-1 Tat, mutational analysis has shown that the Arg-rich basic region (47–59) is required for binding to TAR RNA [65]. The basic domain located in regions 47–59 of the Tat forms an alpha helix during Tat-TAR binding [51]. Modifications in amino acid sequence on the functional groups of Tat proteins have also been shown to affect hydrogen bonding to TAR RNA, lowering the binding affinity by up to 20-fold [66]. The amino acid substitution S46F in the Tat core region could lead to a conformational change to Tat resulting in more hydrogen bond interactions than in the wild-type making it a highly potent transactivator [67]. In addition, the K51R mutation was shown to make Tat more flexible in this location, giving it a direct hydrogen interaction which is more non-rigid than in the wild-type [68]. Thus, from this knowledge, Tat mutations may affect Tat-TAR interaction and the rate of transcription and ultimately the rate of viral replication.

Although many studies have investigated Tat mutations in Tat-TAR interactions [69–72], to our best knowledge, no study has summarised findings for which Tat amino acids and/or regions are the most important in Tat-TAR binding affinity and if subtype-specific Tat sequence variation influences Tat-TAR binding. Therefore, the primary aims of this scoping review were to determine; (1) the regions of the Tat protein that may be involved in TAR binding, (2) the key Tat amino acids involved in TAR binding; and (3) if Tat subtype-specific variation influences TAR binding. The secondary aims were to determine (1) the value of undertaking a full systematic review and meta-analysis; and (2) the extent of the available evidence by reviewing all literature on this topic to date. Findings from this study may help further develop our understanding of the subtype-specific Tat function.

## Methods

### Study design

This is a descriptive and narrative scoping review aimed at synthesising the extant literature of basic/fundamental studies investigating HIV-1 Tat-TAR binding.

### Eligibility criteria

For inclusion, studies needed to investigate Tat-TAR interaction/binding to identify key Tat amino acid regions and/or specific amino acids which may influence Tat-TAR binding affinity. Only studies investigating the HIV-1 Tat protein and/or Tat-derived peptides were included. Investigations of all other Tat proteins (HIV-2, bovine Tat etc.) were excluded. Studies that investigated HIV-1 Tat protein amino acid variation in transactivation assays with no direct binding assays were also excluded. Therefore, the studies had to investigate Tat-TAR binding with a relevant binding assay ((e.g., electrophoretic mobility shift assay (EMSA), surface plasmon resonance (SPR) etc.)) to be included. To ensure uniformity in the included studies, only those studies that reported dissociation constants (Kd) as a measure for the binding affinity between Tat-TAR were included. Studies not published in English were excluded and no data was extracted. Review articles, thesis, conference proceedings and book chapters were also excluded.

### Data sources

We electronically searched for publications in PubMed, Scopus and Web of Science databases based on all studies published until 28/11/2022. The search strategy was executed without publication date limitations. The full search criteria for each database are included in the Additional file 1: File S1. The following search terms were applied to PubMed: (HIV [mh] OR HIV [tw] OR Acquired Immunodeficiency Syndrome [mh] OR “acquired immunodeficiency syndrome” [tw] OR AIDS [tw]) AND (Gene Products, Tat [mh] OR transactivation of transcription [tw] OR Tat [tw]) AND (transactivating response region [tw] OR TAR [tw] OR Tat-TAR [tw] OR HIV Long Terminal Repeat [mh] OR Tat-TAR binding [tw]).

**Fig. 3:**
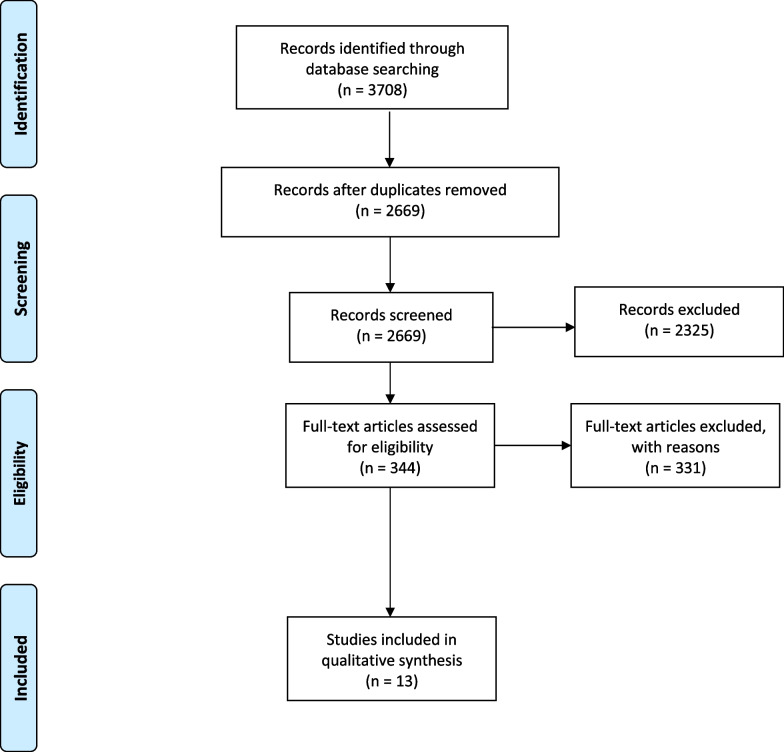
Preferred Reporting Items for Systematic Reviews and Meta-Analyses (PRISMA) flow diagram for results of the search strategy

In addition, we also (1) reviewed reference sections of eligible articles and manually searched for relevant publications and (2) consulted with the corresponding authors of the included studies. This search strategy and the retrieved articles are shown in Fig. 3.

### Data selection

All articles were retrieved and loaded onto a single database using a reference manager (EndNote X9, Clarivate, PA, USA). Two authors, PTG and MEW independently identified studies meeting the inclusion criteria. Where there was a discrepancy in article inclusion/exclusion, this was discussed amongst all authors, and a decision was made regarding its suitability.

### Quality assessment

The quality of the included studies was assessed by PTG and MEW and a kappa statistic was calculated. The quality criterion has been adopted from the CRIS Guidelines (Checklist for Reporting In-vitro Studies) [73]. Here we have amended the CRIS Guidelines by implementing a Likert scale [74] to provide a quantitative measure of study quality. The CRIS guidelines suggest that several areas need to be addressed to promote the quality and transparency of evidence. However, we have selected those areas that may have influenced the findings in the included studies, and these included the reporting of (1) sample size calculation; (2) sample preparation and handling; and (3) statistical analysis. Therefore, these areas were addressed with the following questions (1) were the sample sizes clearly defined; (2) was there a detailed explanation about sample preparation and sample handling to ensure replication of the experimentation; and (3) have appropriate statistical analysis been applied to address the research question. Each question was rated for 0 = no, 1 = partly and 2 = yes. Studies that addressed all the above questions and had a total rating of 6 were classified as high quality. Studies with a rating between 3 and 5 were considered intermediate-quality and less than 3 was low quality.

## Results

### Study characteristics

Using this criterion and search strategy (section “Eligibility criteria”, “Data sources”), 3708 articles were extracted. Duplicates (n = 1039) were removed, resulting in 2669 studies. Thereafter, abstracts and titles were screened and a total of 2325 studies were excluded which comprised of:

- Review articles/thesis/book chapters/conference proceedings (n = 618).
- Studies not investigating HIV-1 Tat-TAR interactions in general (n = 1493).
- Studies not published in English (n = 10).
- Studies not investigating HIV-1 in general (n = 175).
- Studies investigating HIV-2 (n = 23).
- Studies investigating Tat-TAR interactions, but not investigating Tat amino acids/regions influencing interactions (n = 6).

Full-text articles assessed for eligibility were done for 344 studies, and an additional n = 331 were excluded:

- Review articles/book chapters/conference proceedings (n = 21).
- Studies not investigating HIV-1 Tat-TAR interactions in general (n = 14).
- Studies not investigating HIV-1 in general (n = 6).
- Studies investigating Tat-TAR interactions, but not investigating Tat amino acids/regions influencing interactions (n = 217).
- Studies investigating Tat transactivation but not reporting findings for Tat-TAR binding affinities (n = 12).
- Studies investigating Tat-TAR interactions, but only reporting TAR mutants (n = 5). These studies were excluded because the focus of the manuscript was to identify the possible variation of the Tat amino acids only and not the variation of TAR.
- Studies investigating Tat-TAR interactions but with the addition of post-translational modifications of Tat amino acids and use of unnatural Tat peptides (e.g., acetylation, methylation etc.) (n = 29). These studies were excluded as we wanted to determine the Tat amino acids involved in TAR binding interaction and affinity without the potential confounding influence of Tat modifications on these interactions.
- Studies investigating Tat-TAR interaction in the presence of additional interacting partners (e.g., neomycin) (n = 4). These studies were excluded as we wanted to determine the Tat amino acids involved in TAR binding interaction and affinity without the potential confounding influence of additional interacting partners on these interactions.
- Studies not investigating HIV-1 Tat proteins (e.g., Bovine Tat) (n = 4).
- Studies not reporting Kd values (n = 14).
- In silico studies only (n = 5).

Using this criterion (section “Study design”– “Data sources”), a total of 13 fundamental studies were included for data extraction (Fig. 3).

### Quality assessment

The quality of the included studies was assessed by PTG and MEW independently and the inter-rater reliability was assessed. The Kappa statistic for inter-rater agreement and reliability was 0*.683*, indicating substantial agreement [75]. The majority of articles were rated as intermediate (62%) followed by high quality (38%). No study was rated as low quality (Additional file 2: Table S1A and B).

### Regions of the Tat protein that influence Tat-TAR binding

Certain regions of the Tat protein may be important for TAR binding. The majority of studies used Tat peptides covering the basic domain (47–58) for investigating Tat-TAR interactions (Table 1). As a baseline, studies investigated binding affinity to TAR using Tat peptides with only the basic domain (47–58). Thereafter, amino acids were added to either domain individually or both the N-terminal and C-terminal domain together and thereafter binding affinity was measured. Of all studies, four studies investigated the addition of amino acids to the Arg-rich domain (47–58) [69, 76–78] (Table 1). It is relevant to note that this region has been widely investigated due to its confirmed function in Tat-TAR binding [79], and Tat mutations found in this region have influenced Tat-RNA interaction in vivo [80]. A study found that the addition of only N-terminal domain amino acids resulted in no difference in binding affinity to TAR [69]. This is an interesting finding as others have found the N-terminal domain to be involved in TAR interaction [35, 36]. However, several studies reported that the addition of C-terminal Tat amino acids resulted in increased binding affinity to TAR (> 50% increase in binding affinity) [69, 77, 78]. The addition of both N and C terminal Tat amino acids increased binding affinity to TAR (52–85%) [76] (Table 1). A similar trend was noted when amino acids were removed from full-length Tat proteins (1–86) [36, 81]. Based on these findings, it may be hypothesized that the Tat regions outside the basic domain may be important for Tat-TAR interactions, however, this warrants further investigation. With the removal of N and C terminal amino acids resulting in peptides spanning the region of amino acids 37–72 or 48–72, it was reported that binding affinity was decreased (> 1000%) [36]. Interestingly, one study found that removing the N-terminal amino acids 1–29 resulted in no binding of TAR [81] (Table 1). The significance of these regions/domains of the Tat protein in TAR binding is discussed further in the “Discussion” section.

**Table 1:** The influence of multiple Tat amino acid deletions/insertions on Tat-TAR binding affinity

Tat amino acid region	Addition/remove	Binding affinity (Kd)	Percentage increase/decrease in binding affinity	Technique	References
47–58	Addition of N-terminal residues FITKALGIS (38–58)	6 × 10^−9^ M	No difference	EMSA	[69]
	Addition of C-terminal “SGQ” (48–62)	3 × 10^−9^ M	50% increase	EMSA	[69]
	Addition of N-terminal “FTKKALGIS” and C-terminal “EDSQTHQVSLPKQ” (38–72)- N terminal rhodamine (rho)	1.0 × 10^–9^	52% increase	Fluorescence resonance energy transfer (FRET)	[76]
	Addition of N-terminal “FTKKALGIS” and C-terminal “EDSQTHQVSLPKQC” (38–72)- C terminal rho	3.1 × 10^–10^	85% increase	FRET	[76]
	Addition of C-terminal “PQGSQT” (47–64)	8.7 × 10^–7^ M	66% increase	Gel shift assay, CD Spectroscopy and SPR	[77]
	Addition of C-terminal “PQGSQTHR” (47–66)	9.3 × 10^–7^	64% increase	Gel shift assay, CD Spectroscopy and SPR	[77]
	Addition of C-terminal “QTHQVSLSKQ” (48–72)	5 × 10^−9^ M	Increased	Gel retardation assays	[78]
	Addition of C-terminal “QTHQVSLSKQPTSQPRGDPTGPKE” (48–86)	5 × 10^−9^ M	Increased	Gel retardation assays	[78]
1–86	Removal of N-terminal residues 1–29	No binding	No binding	EMSA and SPR	[81]
	Removal of N-terminal residues 1–36 and C-terminal residues 73–86 (Peptide 37–72)	8.4 × 10^–8^	1153.73% decrease	EMSA and dual-label filter binding assay	[36]
	Removal of N-terminal residues 1–42 and C-terminal residues 73–86 (Peptide 43–72)	7.6 × 10^–8^	1034% decrease	EMSA and dual-label filter binding assay	[36]
	Removal of N-terminal residues 1–47 and C-terminal residues 73–86 (Peptide 48–72)	3.0 × 10^–7^	4377% decrease	EMSA and dual-label filter binding assay	[36]
	Removal of N-terminal residues 1–31 and C-terminal residues 73–86 (Peptide 32–72)	2.1 × 10^–7^	3034% decrease	EMSA and dual-label filter binding assay	[36]
	Removal of N-terminal residues 1–48 (Peptide 49–86)	5.3 × 10^–8^	691% decrease	EMSA and dual-label filter binding assay	[36]

### Multiple and single Tat amino acid substitutions that may influence Tat-TAR binding

Of all included studies, six studies investigated Tat-TAR binding and the influence of multiple amino acid substitutions (Table 2) [69, 70, 72, 82–84]. None of the studies investigated naturally occurring Tat subtype-specific mutations. The majority of studies introduced either Lysine (Lys) or Alanine (Ala) to investigate Tat-TAR binding within the cysteine-rich domain (22–38) or basic Arg domain (47–58). One study reported a double substitution in the cysteine-rich domain (22–38), in particular, C34S and C37W which resulted in a lower binding affinity to TAR (19.7%) [70]. All other studies investigated two to eight Tat substitutions within the basic domain (47–58) (Table 2). The substitution of only Ala resulted in the largest percentage decrease in binding affinity across all studies (≥ 1566%) [69, 82]. Because substitution with Ala removes all side-chain atoms past the β-carbon, the effects of individual Ala mutations can be used to infer the roles of wild-type individual side chains and ultimately elucidate the role of particular amino acids in Tat-TAR binding [85]. Studies introducing two to four Lys amino acids only within the basic domain (47–58) resulted in smaller decreases in binding affinity (25%-116%) [69, 83, 84]. Studies reporting five to seven Lys substitutions within the basic domain (47–58) reported much higher decreases in binding affinity (> 1000%) compared to substitutions of two to four Lys amino acids (Table 2). Two studies substituted a combination of multiple Ala, Lys and Arg amino acids within the Tat protein, and this resulted in either no binding [82] or a higher decrease in binding affinity (≥ 250%) compared to wild-type Tat peptides used in the respective studies [82](Table 2). It is also relevant to note that different binding interactions and affinities were recorded by different techniques including Nuclear Magnetic Resonance Spectroscopy (NMR) and Fluorescence spectroscopy [70, 72], Fluorescence resonance energy transfer (FRET), Matrix-assisted laser desorption/ionization-Time of Flight Mass Spectrometry (MALDI-TOFMS) and Fluorescence binding assay [82], EMSA [69, 84], Gel electrophoresis and circular dichroism (CD) [83] and Electron paramagnetic resonance spectroscopy (EPR) [72] (Table 2).

**Table 2:** The influence of multiple Tat amino acid substitutions on Tat-TAR binding affinity

Tat amino acid region	Mutation	Binding affinity (Kd)	Percentage increase/decrease in binding affinity	Technique	References
30–46	C34S, C37W	7.16 × 10^−8^ M	19.7% decrease	Nuclear Magnetic Resonance Spectroscopy (NMR) and Fluorescence spectroscopy	[70]
47–58	R49A, Q54A	1.7 × 10^−7^ M	142% decrease	Fluorescence resonance energy transfer (FRET), MALDI-TOFMS and Fluorescence binding assay	[82]
	R52A, R53A	> 100 X 10^−9^ M	> 1566% decrease	Electrophoretic mobility shift assay (EMSA)	[69]
	R55A, R56A	> 100 X 10^−9^ M	> 1566% decrease	EMSA	[69]
	R55K, R56K	4 × 10^−9^ M	33% increase	EMSA	[69]
	R52K, R53K	13 X 10^−9^ M	116% decrease	EMSA	[69]
	R52A, R55A	> 100 X 10^−9^ M	> 1566% decrease	EMSA	[69]
	R52A, R56A	> 100 X 10^−9^ M	> 1566% decrease	EMSA	[69]
	R53A, R55A	> 100 X 10^−9^ M	> 1566% decrease	EMSA	[69]
	R53A, R56A	> 100 X 10^−9^ M	> 1566% decrease	EMSA	[69]
	K50A, K51A, Q54A	2.9 × 10^−6^ M	4042% decrease	FRET, MALDI-TOFMS and Fluorescence binding assay	[82]
	R49K, R55K, R56K, R57K	5 × 10^−10^ M	25% decrease	Gel electrophoresis and circular dichroism (CD)	[83]
	R49A, K50A, K51A, Q54A	1.8 × 10^−5^ M	21,328.6% decrease	FRET, MALDI-TOFMS and Fluorescence binding assay	[82]
	R49K, R53K, R55K, R56K, R57K	9 × 10^−10^ M	125% decrease	Gel electrophoresis and CD	[83]
	R49K, Q54K, R55K, R56K, R57K	1.8 × 10^−9^ M	350% decrease	Gel electrophoresis and CD	[83]
	R49K, R52K, R53K, R56K, R57K	> 5 × 10^−8^ M	> 12,400% decrease	Gel electrophoresis and CD	[83]
	R49K, R52K, R53K, R55K, R57K	> 1 × 10^−7^ M	> 24,900% decrease	Gel electrophoresis and CD	[83]
	R49K, R53K, Q54K, R55K, R56K, R57K	7 × 10^−10^ M	75% decrease	Gel electrophoresis and CD	[83]
	R49K, R52K, R54K, R55K, R56K, R57K	3.5 × 10^−9^ M	775% decrease	Gel electrophoresis and CD	[83]
	R49K, R52K, R53K, R55K, R56K, R57K	3.2 × 10^–8^	1428% decrease	EMSA and Anisotropy assay	[84]
	R49A, K50A, Q54A, K51R, R53A, Q54R, R57A	6.6 × 10^−5^ M	94,185% decreased	FRET, MALDI-TOFMS and Fluorescence binding assay	[82]
	R49A, K50A, R51R, R53A, Q54R, R56A, R57A	No binding	No binding	FRET, MALDI-TOFMS and Fluorescence binding assay	[82]
	R49A, K50A, K51R, R53A, Q54R, R55A, R56A, R57A	No binding	No binding	FRET, MALDI-TOFMS and Fluorescence binding assay	[82]
	G48K, R49K, R52K, R53K, Q54K, R55K, R56K, R57A	1.5 × 10^–6^ M	275% decrease	Electron paramagnetic resonance spectroscopy (EPR)	[72]

Several studies investigated the influence of single amino acid substitutions in Tat-TAR binding (Table 3). In particular, four studies [69, 71, 82, 86] investigated single amino acid substitutions (Table 3). None of the studies investigated naturally occurring Tat subtype-specific mutations but rather substituted wild-type amino acids with Ala. The majority of studies investigated the basic domain (47–58) as this is the known interacting partner for TAR. The largest decreases in binding affinities (> 1900%) were recorded for the Tat substitutions K49A, K50A, K51A, K53A, K54A and K56A (Table 2). When Lys was mutated to Ala, a significant decrease in binding affinity was observed. In contrast to this, when Arg or Glutamine (Gln) was mutated to Ala, smaller decreases in binding affinity were observed. An example of this is when Arg was mutated (i.e., R5K, R52A, R53A, R55A, R56A), and the binding affinity showed a small decrease (40–70%) compared to when Lys was mutated (i.e., K49A, K50A, K51A, K53A, K54A and K56A) which resulted in a larger decrease in binding affinity (> 1900%) (Table 3). Interestingly, one study reported that the Q54A substitution resulted in an increased binding affinity compared to the wild-type Tat peptide (20% increase) [69] (Table 3). These suggest that the binding affinity of Tat-TAR may be influenced by which amino acids are present in the wild-type Tat protein (Lys, Arg or Gln). Further, this may suggest that, compared to Arg amino acids in the basic domain (47–58), Lys amino acids may be greater contributors to the binding affinity to TAR. It is relevant to note that the binding interaction and affinities were recorded by different techniques SPR [86], Absorption spectroscopy, Gel shift assays, CD Spectroscopy [71], Electrophoretic mobility shift assay (EMSA) [69] and Fluorescence resonance energy transfer (FRET), MALDI-TOFMS, Fluorescence binding assay [82] (Table 3). Even though different techniques were employed across these four studies, a consistent trend was noted that when wild-type amino acids were substituted, a lower binding affinity was observed, confirming the notion that amino acids within the basic domain (47–58) are important for TAR binding. A summary of all key findings is given in Table 4.

**Table 3:** The influence of single point Tat amino acid substitutions on Tat-TAR binding affinity

Tat amino acid position	Mutation	Binding affinity	Percentage increase/decrease in Tat-TAR binding affinity	Technique	References
5	R5K	6.7 × 10^−7^ M	70% decrease	Surface plasmon resonance (SPR)	[86]
49	K49A	4 × 10^−6^ M	1900% decrease	Absorption spectroscopy, Gel shift assays, CD Spectroscopy	[71]
50	K50A	2 × 10^–5^ M	9900% decrease	Absorption spectroscopy, Gel shift assays, CD Spectroscopy	[71]
51	K51A	4 × 10^−6^ M	1900% decrease	Absorption spectroscopy, Gel shift assays, CD Spectroscopy	[71]
52	R52A	10 × 10^−9^ M	40% decrease	Electrophoretic mobility shift assay (EMSA)	[69]
53	R53A	12 X 10^−9^ M	50% decrease	EMSA	[69]
	K53A	4 × 10^−6^ M	1900% decrease	Absorption spectroscopy, Gel shift assays, CD Spectroscopy	[71]
54	Q54A	5 X 10^−9^ M	20% increase	EMSA	[69]
		8 × 10^−8^ M	12.5% decrease	Fluorescence resonance energy transfer (FRET), MALDI-TOFMS, Fluorescence binding assay	[82]
	K54A	2 × 10^–5^	1900% decrease	Absorption spectroscopy, Gel shift assays, CD Spectroscopy	[71]
55	R55A	12 × 10^−9^ M	50% decrease	EMSA	[69]
56	R56A	12 × 10^−9^ M	50% decrease	EMSA	[69]
	K56A	4 × 10^−6^ M	1900% decrease	Absorption spectroscopy, Gel shift assays, CD Spectroscopy	[71]

**Table 4:** Key findings from studies investigating Tat-TAR binding affinities

Reference	Binding technique	Amino acids (Being investigated)	Mutation introduced	In silico, In vitro/In vivo	Key findings
[86]	Surface Plasmon Resonance (SPR)	Tat peptides Wild type: RVRTRKGRRIRIPP Tat peptide 1 RVRTKKGRRIRIPP	Tat peptide 1: R5K	In vitro	1. Dissociation constant (K_d_) values of Tat-TAR binding: Tat wild type = 0.2 × 10^−7^ M Tat peptide 1 = 6.7 × 10^−7^ M 2. R5K mutation in Tat peptide 1 can destabilize Tat-TAR interaction, reducing the rate of Tat release from TAR, decreasing overall affinity of Tat for TAR 3. When TAR binds to Tat peptide 1 the K_d_ value increased significantly (+ 15-fold) compared to the Tat wild type
[69]	Electrophoretic mobility shift assay (EMSA)	Tat peptides (47–58) Tat wild type: YGRKKRRQRRRP Tat peptide 1: YGRKKARQRRRP Tat peptide 2: YGRKKRAQRRRP Tat peptide 3: YGRKKRRARRRP Tat peptide 4: YGRKKRRQARRP Tat peptide 5: YGRKKRRQRARP Tat peptide 6: YGRKKAAQRRRP Tat peptide 7: YGRKKRRQAARP Tat peptide 8: YGRKKRRQKKRP Tat peptide 9: YGRKKKKQRRRP Tat peptide 10: YGRKKARQARRP Tat peptide 11: YGRKKARQRARP Tat peptide 12: YGRKKRAQARRP Tat peptide 13: YGRRRRAQRARP Tat peptide 14: FITKALGISYGRKKRRQRRRP Tat peptide 15: SGQPPRRRQRRKKRG Tat peptide 16: YRKRRQRRGKRP Tat peptide 17: YRKRGRQRRKRP	Tat peptide 1: R52A Tat peptide 2: R53A Tat peptide 3: Q54A Tat peptide 4: R55A Tat peptide 5: R56A Tat peptide 6: R52AR53A Tat peptide 7: R55AR56A Tat peptide 8: R55KR56K Tat peptide 9: R52KR53K Tat peptide 10: R52AR55A Tat peptide 11: R52AR56A Tat peptide 12: R53AR55A Tat peptide 13: R53AR56A Tat peptide 14: Addition of N-terminal “FITKALGIS” Tat peptide 15: Addition of C-terminal “SGQ” Tat peptide 16: G48R, K50R, R52Q, Q54R, R55K, R56K, P58G Tat peptide 17: G48R, K50R, K51G, R53Q, Q54R, R56K	In vitro	1. K_d_ values of Tat-TAR binding: Tat wild type = 6 X 10^−9^ M 2. When single mutations were introduced into Tat, the following Kd values were observed: Tat peptide 1 = 10 × 10^−9^ M Tat peptide 2 = 12 X 10^−9^ M Tat peptide 3 = 5 X 10^−9^ M Tat peptide 4 = 12 × 10^−9^ M Tat peptide 5 = 12 × 10^−9^ M 3. When Double mutations were introduced in Tat, the following K_d_ values were observed: Tat peptide 8 = 4 × 10^−9^ M Tat peptide 9 = 13 X 10^−9^ M Tat peptide 6, Tat peptide 7, Tat peptide 10, Tat peptide 11, Tat peptide 12, Tat peptide 13 = K_d_ = > 100 X 10^−9^ M 4. The Tat mutation Q54A in Tat peptide 3 had no significant effect on Tat-TAR binding 5. The Tat peptide 14 had a binding affinity of 6 × 10^−9^ M to TAR whereas the Tat peptide 15 had a binding affinity of 3 × 10^−9^ M Tat peptide 16 = 4 × 10^−9^ M Tat peptide 17 = 3 × 10^−9^ M 6. The Arginine (R)-rich region 47–58 of the Tat protein binds to 3’ nucleotide bulge of TAR-RNA 7. Substituting pairs of positions R52AR53A (Tat peptide 6), R55AR56A (Tat peptide 7), R52AR55A (Tat peptide 10), R52AR56A (Tat peptide 11), R53AR55A (Tat peptide 12), R53AR56A (Tat peptide 13) reduced binding affinity to TAR > 20 fold 8. Substituting a single R on Tat positions R52A (Tat peptide 1), R53A (Tat peptide 2), R55A (Tat peptide 4), R56A (Tat peptide 5) reduced binding to TAR by twofold 9. Therefore, double substitutions of R with A in the 47–58 region resulted in the highest K_d_ values, showing the lowest Tat-TAR binding affinity 10. Multiple double substitutions were made on Tat peptide 16 – G48R, K50R, R52Q, Q54R, R55K, R56K, P58G Tat peptide 17- G48R, K50R, K51G, R53Q, Q54R, R56K Tat peptides 15, 16, 17 had lower K_d_ values compared to Tat wild type
[76]	Fluorescence resonance energy transfer (FRET)	Tat peptides (47–58 and 38–72) Tat 38–72: FTKKALGISYGRKKRRQRRRAPEDSQTHQVSLPKQ Tat peptide 1: N-terminus rh-Tat YGRKKRRQRRRP Tat peptide 2: N-terminus rh-Tat FTKKALGISYGRKKRRQRRRAPEDSQTHQVSLPK Tat peptide 3: FTKKALGISYGRKKRRQRRRAPEDSQTHQVSLPKQC C terminus rh-Tat (38–72)	Comparing Tat fragments/regions	In vitro	1. K_d_ values of binding to TAR RNA 2. Tat peptide 1 = 2.1 × 10^–9^ (± 0.2 × 10^–10^) M; 3. Tat peptide 2 = Rh-Tat (38–72) = 1.0 × 10^–9^(± 0.1 × 10^–10^) M 4. Tat peptide 3 = Tat (38–72) C-Rh = 3.1 × 10^–10^ (± 0.2 × 10^–10^) M, and 5. The reason for Tat peptides consisting of 38–72 having higher binding affinity is because the amino acids from the core region makes the most contacts with TAR RNA and stabilize the structure of the TAR-Tat complex to enhance the affinity and specificity of Tat for TAR 6. The reason for the *K*_d_ value of Tat peptide 2 being threefold greater than Tat peptide 3 was that in Tat peptide 3, rhodamine moiety is relatively far from the basic binding region of Tat and will not influence binding affinity significantly
[81]	EMSA and SPR	Tat peptide 1 Tat full length (1–86) EPVDPRLEPWKHPGSQPKTACTTCYCKKCCFHCQVCFTTKALGISYGRKKRRQRRRPPQGSQTHQVSLSKQPTSQPRGDPTGPKE Tat peptide 2 Biot-Tat (1–86): EPVDPRLEPWKHPGSQPKTACTTCYCKKCCFHCQVCFTTKALGISYGRKKRRQRRRPPQGSQTHQVSLSKQPTSQPRGDPTGPKE Tat peptide 3 Biot-Tat (30–86): CFHCQVCFTTKALGISYGRKKRRQRRRPPQGSQTHQVSLSKQPTSQPRGDPTGPKE	Comparing Tat fragments/regions	In vitro/in silico	1. Tat peptide 3 (30–86) did not bind to TAR 2. This suggested that if the N terminus of Tat is deleted, no binding to TAR will take place 3. Tat peptide 2 (1–86) bound to TAR at a K_d_ of 1.85 × 10^−9^ M 4. Tat peptide 1 (1–86) bound to TAR at a K_d_ of 6.71 × 10^−9^ M
[36]	EMSA and dual-label filter binding assay	Full length Tat, HIV-1_BRU_ (1–86) Tat peptides (32–86): Tat peptide 1 (37–72): CFTTKALGISYGRKKRRQRRRPPQGSQTHQVSLSKQ Tat peptide 2 (43–72): LGISYGRKKRRQRRRPPQGSQTHQVSLSKQ Tat peptide 3 (48–72): GRKKRRQRRRPPQGSQTHQVSLSKQ Tat peptide 4 (32- 72): FHCQVCFTTKALGISYGRKKRRQRRRPPQGSQTHQVSLSKQ Tat peptide 5 (49–86): RKKRRQRRRPPQGSQTHQVSLSKQPTSQSRGDPTGPKE Tat peptide 6 (32–62): FHCQVCFTTKALGISYGRKKRRQRRRPPQGS	Comparing Tat fragments/regions	In vitro	1. K_d_ values for full length Tat peptides to TAR were as follow Full length Tat: 6.7 × 10^−9^ M, Tat peptide 1: 8.4 × 10^–8^ (± 1.3 × 10^–8^) M, Tat peptide 2: 7.6 × 10^–8^ (± 8 × 10^–9^) M, Tat peptide 3: 3.0 × 10^–7^ (± 2.0 × 10^–8^) M, Tat peptide 4: 2.1 × 10^–7^ (± 2.1 × 10^–8^) M, Tat peptide 5: 5.3 × 10^–8^ (± 2 × 10^–9^) M 2. No findings were reported for Tat peptide 6 3. Tat affinity and specificity for TAR RNA are increased when core region (40–48) was present in the peptide, with K41 playing a key role in TAR recognition 4. Peptides which contain only the basic region & C terminal bind to TAR RNA weakly and non-specifically
[72]	Electron paramagnetic resonance spectroscopy (EPR)	Tat peptides (47–57) Tat wild type: YGRKKRRQRRR Tat peptide 1 YKKKKRKKKKA	Tat peptide 1: G48K, R49K, R52K, R53K, Q54K, R55K, R56K, R57A	In vitro/In silico	1. K_d_ values for Tat peptides binding TAR were as follow: Tat wild type -TAR = 4.0 × 10^–7^ M and Tat peptide 1-TAR = 1.5 × 10^–6^ M 2. The Presence of R52 in both peptides has similar binding affinity to TAR RNA 3. This suggest that other amino acids in wild type peptides account for difference in binding affinity and the development of a more rigid complexes between the wild type and peptide 1
[84]	EMSA Anisotropy assay	Tat peptides (47–57) Tat wild type peptide = YGRKKRRQRRR Tat peptide 1 = YGKKKKKQKKK	Tat peptide 1: R49K, R52K, R53K, R55K, R56K, R57K	In vitro	1. K_d_ values for Tat-TAR binding was as follow: Tat wild type peptide = 2.1 × 10^–9^ (± 8 × 10^–10^) M, Tat peptide 1 = 3.2 × 10^–8^ (± 4 × 10^–9^) M 2. Tat affinity for RNA depends on side chain of R in basic region – and cationic side chains providing a polyelectrolyte-like affinity 3. A cluster of cationic residues may also provide a polyelectrolyte affinity of Tat for TAR
[83]	Gel electrophoresis Circular dichroism (CD)	Tat peptide (49–57) Tat wild type—RKKRRQRRR Tat peptide 1-KKKRKQKKK Tat peptide 2- KKKRKKKKK Tat peptide 3- KKKKRQKKK Tat peptide 4- KKKRRQKKK Tat peptide 5—KKKRRKKKK Tat peptide 6- KKKKKQRKK Tat peptide 7- KKKKKQKRK	Tat peptide 1 = R49K, R53K, R55K, R56K, R57K Tat peptide 2 = R49K, R53K, Q54K, R55K, R56K, R57K Tat peptide 3 = R49K, R52K, R54K, R55K, R56K, R57K Tat peptide 4 = R49K, R55K, R56K, R57K Tat peptide 5 = R49K, Q54K, R55K, R56K, R57K Tat peptide 6 = R49K, R52K, R53K, R56K, R57K Tat peptide 7 = R49K, R52K, R53K, R55K, R57K	In vitro	1. K_d_ values for Tat-TAR binding were as follow: Tat wild type = 4 × 10^−10^ M, Tat peptide 1 = 9 × 10^−10^ M, Tat peptide 2 = 7 × 10^−10^ M, Tat peptide 3 = 3.5 × 10^−9^ M, Tat peptide 4 = 5 × 10^−10^ M, Tat peptide 5 = 1.8 × 10^−9^ M, Tat peptide 6 = > 5 × 10^−8^ M and Tat peptide 7 = > 1 × 10^−7^ M 2. Therefore, Tat wild type had the highest binding affinity 3. Tat peptide 7 had the lowest binding affinity and this may be as a result of the absence of arginine in this amino acid sequence
[82]	Fluorescence resonance energy transfer (FRET) MALDI-TOFMS Fluorescence binding assay	Tat peptides (46–61) Tat wild type: 46AAARKKRRQRRRAAA60 Tat peptide 1: 46AAARKKRRARRRAAA61 Tat peptide 2: 46AAAAKKRRARRRAAA61 Tat peptide 3: 46AAARAARRARRRAAA61 Tat peptide 4: 46AAAAARRRRRAAAAAA61 Tat peptide 5: 46AAAAARRARRRAAAAA61 Tat peptide 6: 46AAAAARRARRAAAAAA61 Tat peptide 7: 46AAAAARRARAAAAAAA61 Tat peptide 1: 46AAAAARRARAAAAAAA61 Tat peptide 2: 46AAAAARRARRAAAAAA61 Tat peptide 3: 46AAAAARRARRRAAAAA61 Tat peptide 4: 46AAAAARRRRRAAAAAA61 Tat peptide 5: 46AAARAARRARRRAAA61 Tat peptide 6: 46AAAAKKRRARRRAAA61 Tat peptide 7: 46AAARKKRRARRRAAA61	Tat peptides Tat peptide 1—Q54A Tat peptide 2- R49A, Q54A Tat peptide 3—K50A, K51A, Q54A Tat peptide 4—R49A, K50A, K51A, Q54A, Tat peptide 5 – R49A, K50A, Q54A, K51R, R53A, Q54R, R57A Tat peptide 6—R49A K50A, R51R, R53A, Q54R. R56A, R 57A Tat peptide 7 – R49A, K50A, K51R, R53A, Q54R R55A, R56A, R57A	In vitro	1. K_d_ Values between Tat peptides and TAR were as follow: Tat wild type: 7 × 10^−8^ M; Tat peptide 1 (K_d_ = 8 × 10^−8^ M); Tat peptide 2 (K_d_ = 1.7 × 10^−7^ M); Tat peptide 3 (K_d_ = 2.9 × 10^−6^ M); Tat peptide 4 (K_d_ = 1.8 × 10^−5^ M); Tat peptide 5 (K_d_ = 6.6 × 10^−5^ M); Tat peptide 6 = No binding, Tat peptide 7 = No binding, 2. The Tat wild type peptide with K_d_ 7 × 10^−8^ M had the highest affinity for TAR
[70]	Nuclear Magnetic Resonance Spectroscopy (NMR) Fluorescence spectroscopy	Tat peptide (32–72) Tat wild type YHCQVCFITKGLGISYGRKKRRQRRRPSQGGQTHQDPIPKQ Tat peptide 1: YHSQVWFITKGLGISYGRKKRRQRRRPSQGGQTHQDPIPKQ	Tat peptide 1: C34S, C37W	In vitro	1. K_d_ values of Tat wild type -TAR and Tat peptide 1 – TAR was as follows 2. Tat peptide 1 bound to TAR RNA was 7.16 × 10^−8^ M and Tat wild type – TAR binding = 5.75 × 10^−8^ M 3. By comparing K_d_ values of Tat wild type and Tat peptide 1 the introduced mutations, C34S and C37W in Tat peptide 1 indicate that C34 and C37 in core region are not involved in high affinity to HIV-1 TAR RNA
[71]	Absorption spectroscopy Gel shift assays CD Spectroscopy	Tat peptides (49–55): Tat wild type: KKKRKKKK Tat peptide 1: AKKRKKKK Tat peptide 2: KAKKRKKK Tat peptide 3: KKARKKK Tat peptide 4: KKKRAKK Tat peptide 5: KKKRKAK Tat peptide 6: KKKRKKA	Tat peptide 1: K49A Tat peptide 2: K50A Tat peptide 3: K51A Tat peptide 4: K53A Tat peptide 5: K54A Tat peptide 6: K56A	In vitro	1. K_d_ values for Tat binding TAR were as follow: Tat wild type = 2 × 10^−7^ M, Tat peptide 1 = 4 × 10^−6^ M, Tat peptide 2 = 2 × 10^–5^ M, Tat peptide 3 = 4 × 10^−6^ M, Tat peptide 4 = 4 × 10^−6^ M, Tat peptide 5 = 2 × 10^–5^ and Tat peptide 6 = 4 × 10^−6^ M 2. The specificity of a Tat amino acid sequence in binding to TAR is mediated by the R52 located in the middle of basic region and surrounding positive charges to R52 overall binding affinity to TAR
[77]	Gel shift assay CD Spectroscopy, SPR	Tat peptides Tat peptide wild type YGRKKRRQRRRP Tat peptide 1 YGRKKRRQRRRPPQGSQT Tat peptide 2 YGRKKRRQRRRPPQGSQTHR	Comparing Tat fragments/regions	In vitro	1. The binding of Tat peptides to TAR were as follow: Tat peptide wild type -TAR = 2.6 × 10^–6^ M, Tat peptide 1-TAR = 8.7 × 10^–7^ M, Tat peptide 2-TAR = 9.3 × 10^–7^ M, 2. The kinetic stabilities of Tat peptide 1- TAR and Tat peptide 2 -TAR complexes cannot be differentiated: it may be due to minimal difference between the two peptides being only two (Q and H) amino acids on the C terminal region of the Tat peptide 2 Increased kinetic stability in Tat peptides 1 and 2 – TAR complexes indicates that other amino acid residues outside the basic region contribute to overall binding affinity and kinetic stability of the Tat-TAR complex
[78]	Gel retardation assays High performance liquid chromatography (HPLC)	Tat peptides: Tat peptide 1: RKKRRQRRRPPQGS Tat peptide 2: RKKRRQRRRPPQGSQTHQVSLSKQ Tat peptide 3: RKKRRQRRRPPQGSQTHQVSLSKQPTSQPRGDPTGPKE	Comparing Tat fragments/regions	In vitro	1. Tat peptide 3 – TAR binding was approximately 5 × 10^−9^ M and Tat peptide 2-TAR binding approximately 5 × 10^−9^ M 2. Tat 1- TAR—dissociation occurs very fast and could not be measured 3. TAR sequence and structure can contribute to specific recognition by -COOH terminal domain of Tat 4. Residue -COOH terminal to the basic region are not necessary for binding to TAR but their presence contributes to the kinetic stability of Tat-TAR complex 5. Possible hydrogen bond acceptors on Q54 sidechain and peptide backbones on Tat peptide promote a more specific interaction with TAR RNA

## Discussion

Several findings were highlighted in this review, and these included (1) both N-terminal and C-terminal amino acids outside the basic domain (47–58) may be important in Tat-TAR binding, (2) substitution of wild-type Tat amino acid Lys and Arg within the basic domain (47–58) results in a reduction in binding to TAR and (3) none of the included studies have investigated Tat subtype-specific substitutions and therefore no commentary could be made regarding which subtype may have a higher Tat-TAR binding affinity. Lastly, a full systematic review/meta-analysis would not be able to be conducted due to the heterogeneity of the available studies.

First, the general consensus is that when studying Tat-TAR interactions, Tat peptides encompassing the basic domain (47–58) may be sufficient for such investigations [31]. However, based on the findings reported in the included studies, we propose that the Tat regions outside the basic domain may be important for Tat-TAR interactions, however, this warrants further investigation. Peptide fragments showed significantly reduced affinities for TAR in comparison to the full-length Tat protein [36, 87]. In line with this, transactivation analysis also revealed that Tat 1–86 is 20-fold more active than Tat 1–57 and it indicates the role played by the sequence 57–86 in post-transactivation [88]. The majority of studies included in this review have used Tat-derived peptides instead of full-length Tat proteins. It may be that researchers opt to use Tat peptides in such experiments due to the difficulty of recombinantly expressing full-length Tat proteins and their inherent toxicity to cells [89], or the difficulty in artificially synthesizing full-length Tat peptides due to the high percentage of cysteine amino acids that may be at risk for oxidation [90]. Here we highlight that full-length Tat protein may be more suited for understanding Tat-TAR binding interactions and its potential downstream effects.

Second, we found that the substitution of wild-type Tat amino acids Lys, Arg and Gln with Ala resulted in decreases in binding affinity. This may be because even though the virus evolves at a high rate [91] certain amino acids may be functionally conserved [31, 32] and therefore any substitution from the wild-type will result in reduced binding. The percentage decrease in binding affinity is influenced by which amino acids were present in the wild-type Tat peptide or protein. In particular, Lys substitutions within the basic Arg domain (47–58) resulted in the largest decrease in TAR binding, and this may suggest that HIV-1 subtypes (e.g., subtype A) with a greater number of Lys amino acids within the basic domain may have higher binding affinities to TAR. Similarly, we found that substituting Arg with Ala, also resulted in a decrease in TAR binding, albeit at a smaller percentage compared to that of Lys. Therefore, it is plausible to suggest that a Lys and Arg-rich basic domain (47–58) may have a higher binding affinity to TAR compared to Tat with a lower percentage of these amino acids in the basic domain (47–58). This may be plausible for subtype B compared to subtype C, whereby subtype B has a greater number of Lys and Arg in the basic domain (47–58). In a previous computational study done within our group, it was found that Tat subtype B had a higher affinity for the TAR RNA element compared to Tat subtype C based on a higher docking score of − 187.37, a higher binding free energy value of − 9834.63 ± 216.17 kJ/mol, and a higher number of proteins–nucleotide interactions of 26 [92]. However, this warrants further molecular investigation.

Third, no study has compared subtype-specific Tat variation and its influence on Tat-TAR binding. All mutations introduced were Ala for the purpose of removing the function of particular wild-type amino acid side chains. Computational studies done by our group [92] and others [67] indicated that subtype-specific variation may influence TAR binding, however molecular validation of such findings remains unstudied. Therefore, it is not clear which subtype variation may influence Tat-TAR binding in the basic domain (47–58) specifically, as well as the domains in the Tat protein. It is therefore important that future studies investigate Tat subtype-specific variation in TAR binding with the use of full-length Tat proteins. Findings from such studies may help explain why we see different levels of HIV-1 pathogenesis and prevalence when comparing HIV-1 subtypes.

Lastly, based on the findings highlighted in this review, several recommendations can be made. Studies were heterogeneous in design and therefore a full systematic review and meta-analysis could not be conducted. Having said this, it may be important to develop a pipeline to allow studies in this line of work to be conducted uniformly. Therefore, studies should clearly define sample sizes used to answer the research question, sample preparation and handling and the relevant statistical analysis. This review also highlights all studies in this particular area of research to date, and it is relevant to note that the majority of studies have been conducted before the year 2000 (53%). Considering the advancement of research techniques in the genome sequencing of HIV-1, more recent investigations may be able to provide clearer reporting of findings. Lastly, no study has investigated subtype-specific mutations. Considering that HIV-1 may be considered a chronic disease, in addition to investigating targets to block Tat-TAR interactions, studies should also investigate which amino acids contribute to the level of transcription, disease phenotypes in PLWH and the prevalence of HIV-1.

## Conclusion

The HIV Tat protein binds to TAR which is responsible for the initiation of transcription. Based on the findings reported in this review, we propose that Tat amino acids outside the basic Arg domain (47–58) may be important in Tat-TAR binding and full-length Tat peptides should be used for the investigation of Tat-TAR binding. Further, wild-type Tat containing a higher percentage of Lys and Arg amino acids within the basic domain (47–58) may have a higher binding to TAR. To date, limited studies have investigated subtype-specific variation within the Tat protein and its binding to TAR and therefore, it is not clear which subtype may have the highest binding affinity. Future studies should investigate subtype-specific variation and implement full-length Tat protein in such studies. Findings from such studies will aid in understanding Tat-TAR binding and potential downstream effects. In addition, this may also provide new therapeutic targets to prevent Tat-initiated transcription.

## Supplementary Information

**Additional file 1.** Database search terms.

**Additional file 2: Table S1. A** Quality of assessment of studies conducted by PTG. **B** Quality of assessment of studies conducted by MEW.

## Data Availability

All supporting data (Additional files/tables) is attached to this manuscript.

## Abbreviations

AIDS: Acquired Immune Deficiency Syndrome
Ala: Alanine
Arg: Arginine
ART: Anti-retroviral therapy
ARV: Anti-retroviral drugs
CCL2: C–C motif chemokine ligand 2
CD4: Cluster of differentiation four
CDK9: Cyclin-dependent kinase 9
CD: Circular dichroism
COVID-19: Coronavirus disease 2019
CRIS: Checklist for Reporting In-vitro Studies
DNA: Deoxyribonucleic acid
EMSA: Electrophoretic mobility shift assay
EPR: Electron Paramagnetic Resonance
FRET: Fluorescence resonance energy transfer
Gln: Glutamine
HAND: HIV associated neurocognitive disorder
HIV: Human immunodeficiency virus
IRIS: Immune reconstitution inflammatory syndrome
kDa: Kilodaltons
Lys: Lysine
MALDI-TOFMS: Matrix-assisted laser desorption/ionization-Time of Flight Mass Spectrometry
Nef: Negative regulatory factor
NMR: Nuclear magnetic resonance
PLWH: People living with HIV
Pol II: RNA Polymerase II
P-TEFb: Positive transcriptional elongation factor
RGD: Arginylglycylaspartic acid
Rev: Regulator of expression of virion proteins
SARS-CoV-2: Severe acute respiratory syndrome coronavirus type 2
SPR: Surface plasmon resonance
TAR: Transactivation response element
Tat: Transactivation of transcription
Vif: Viral infectivity factor
Vpr: Viral protein R
Vpu: Virus protein U
