# Supplementary material for: HIV-1 Tat amino acid residues that influence Tat-TAR binding affinity: a scoping review
Source: BMC Infect Dis. 2023 Mar 17;23:164. doi: 10.1186/s12879-023-08123-0 (PMC10020771; doi:10.1186/s12879-023-08123-0)
Supplement: Supplementary file 1 — Additional file 1. Database search terms. [file 12879_2023_8123_MOESM1_ESM.docx]

Search terms

**Search conducted on the 28/11/2022**

**Pubmed (1399) 28/11/2022**

(HIV [mh] OR HIV [tw] OR Acquired Immunodeficiency Syndrome [mh] OR “acquired immunodeficiency syndrome” [tw] OR AIDS [tw]) AND (Gene Products, tat [mh] OR transactivation of transcription [tw] OR Tat [tw]) AND (transactivating response region [tw] OR TAR [tw] OR Tat-TAR [tw] OR HIV Long Terminal Repeat [mh] OR Tat-TAR binding [tw])

**Scopus (1250) 28/11/2022**

(HIV OR Acquired Immunodeficiency Syndrome OR AIDS) AND (Tat OR transactivation of transcription OR Trans-Activator of Transcription) AND (Trans-Activator of Transcription OR transactivating response region OR TAR OR Tat-TAR OR Tat-TAR binding)

**Web of science 1059 28/11/2022**

TS=(HIV OR Acquired Immunodeficiency Syndrome OR" Acquired Immunodeficiency Syndrome" OR AIDS) AND TS=(Tat OR transactivation of transcription OR Trans-Activator of Transcription) AND TS=( Trans-activation response element OR transactivating response region OR TAR OR Tat-TAR OR Tat-TAR binding)

Total: 3708
