# Supplementary material for: HIV-1 Tat amino acid residues that influence Tat-TAR binding affinity: a scoping review
Source: BMC Infect Dis. 2023 Mar 17;23:164. doi: 10.1186/s12879-023-08123-0 (PMC10020771; doi:10.1186/s12879-023-08123-0)
Supplement: Supplementary file 2 — Additional file 2: Table S1. A Quality of assessment of studies conducted by PTG. B Quality of assessment of studies conducted by MEW. [file 12879_2023_8123_MOESM2_ESM.docx]

**Table S1A**: Quality of assessment of studies conducted by PTG

| **Reference** | **Q1** | **Q2** | **Q3** | **Score** | **Rating** |
| --- | --- | --- | --- | --- | --- |
| [86] | 2 | 2 | 2 | 6 | High |
| [69] | 2 | 2 | 2 | 6 | High |
| [76] | 2 | 1 | 1 | 4 | Intermediate |
| [81] | 2 | 2 | 2 | 6 | High |
| [36] | 2 | 2 | 2 | 6 | High |
| [72] | 2 | 1 | 2 | 5 | Intermediate |
| [84] | 2 | 1 | 1 | 4 | Intermediate |
| [83] | 1 | 2 | 2 | 5 | Intermediate |
| [82] | 2 | 1 | 2 | 5 | Intermediate |
| [70] | 2 | 2 | 2 | 6 | High |
| [71] | 2 | 2 | 2 | 6 | High |
| [77] | 2 | 1 | 2 | 5 | Intermediate |
| [78] | 1 | 1 | 1 | 3 | Intermediate |

**Table S1B**: Quality of assessment of studies conducted by MEW

| **Reference** | **Q1** | **Q2** | **Q3** | **Score** | **Rating** |
| --- | --- | --- | --- | --- | --- |
| [86] | 2 | 2 | 2 | 5 | High |
| [69] | 2 | 2 | 2 | 6 | High |
| [76] | 2 | 1 | 2 | 6 | Intermediate |
| [81] | 2 | 2 | 2 | 6 | High |
| [36] | 1 | 1 | 2 | 4 | Intermediate |
| [72] | 1 | 2 | 2 | 5 | Intermediate |
| [84] | 1 | 1 | 2 | 4 | Intermediate |
| [83] | 1 | 2 | 2 | 5 | Intermediate |
| [82] | 1 | 1 | 2 | 4 | Intermediate |
| [70] | 2 | 2 | 2 | 6 | High |
| [71] | 1 | 1 | 2 | 4 | Intermediate |
| [77] | 1 | 1 | 1 | 3 | Intermediate |
| [78] | 1 | 2 | 1 | 4 | Intermediate |

The CRIS guidelines suggest the several areas need to be addressed to promote quality and transparency of evidence. However, we have selected those areas that may have influenced the findings in the included studies, and these could include the reporting of (1) Sample size calculation, (2) Sample preparation and handling and (3) Statistical analysis. Therefore, these areas were addressed with the following questions (1) where the sample sizes clearly defined, (2) was there a detailed explanation about sample preparation and sample handling helps to ensure replication of the experimentation, (3) Have appropriate statistical analysis been applied to dress the research question. A detailed explanation about sample preparation and sample handling help. Each question was rated for 0 = no, 1 = partly and 2 = yes. Studies that addressed all the above questions and had a total rating of 6 were classified as high quality. Studies with a rating between 3 and 5 were considered as intermediate-quality and less than 3 as low quality.
